# Nanostructured Chemoresistive Sensors for Oncological Screening and Tumor Markers Tracking: Single Sensor Approach Applications on Human Blood and Cell Samples

**DOI:** 10.3390/s20051411

**Published:** 2020-03-04

**Authors:** Nicolò Landini, Gabriele Anania, Michele Astolfi, Barbara Fabbri, Vincenzo Guidi, Giorgio Rispoli, Matteo Valt, Giulia Zonta, Cesare Malagù

**Affiliations:** 1 Department of Physics and Earth Sciences, University of Ferrara, Via Saragat 1/C, 44122 Ferrara, Italy; stlmhl@unife.it (M.A.); fbbbbr@unife.it (B.F.); gduvcn@unife.it (V.G.); vltmtt1@unife.it (M.V.); zntgli@unife.it (G.Z.);; 2 Department of Morphology, Surgery and Experimental Medicine, University of Ferrara, Via Luigi Borsari 46, 44121 Ferrara, Italy; ang@unife.it; 3 SCENT S.r.l, Via Quadrifoglio 11, 44124 Ferrara, Italy; 4 Department of Life Sciences and Biotechnology, University of Ferrara, Via Luigi Borsari 46, 44121 Ferrara, Italy; rsg@unife.it

**Keywords:** chemoresistive sensors, blood, cells, tumor, screening, metabolites, oncology

## Abstract

Preventive screening does not only allow to preemptively intervene on pathologies before they can harm the host; but also to reduce the costs of the intervention itself; boosting the efficiency of the NHS (National Health System) by saving resources for other purposes. To improve technology advancements in this field; user-friendly yet low-cost devices are required; and various applications for gas sensors have been tested and proved reliable in past studies. In this work; cell cultures and blood samples have been studied; using nanostructured chemoresistive sensors; to both verify if this technology can reliably detect tumor markers; and if correlations between responses from tumor line metabolites and the screening outcomes on human specimens could be observed. The results showed how sensors responded differently to the emanations from healthy and mutant (for cells) or tumor affected (for blood) samples, and how those results were consistent between them, since the tumoral specimens had higher responses compared to the ones of their healthy counterparts. Even though the patterns in the responses require a bigger population to be defined properly; it appeared that the different macro-groups between the same kind of samples are distinguishable from some of the sensors chosen in the study; giving promising outcomes for further research.

## 1. Introduction

The demand for reliable devices to detect tumor biomarkers in the human body is constantly increasing. The reasons behind this lay on the advantages of early intervention on pathologies, allowing for a greater chance of healing and survival for the patients compared to taking action in the malignant state of a neoplasia not diagnosed in time. Consequently, the expenses for the National Health Systems would drop consistently while the efficiency of intervention from physicians and surgeons would increase, causing a reduction in the amount of malignant and terminal cases.

Chemoresistive semiconductor sensors, fast responding devices commonly used for pollution and alimentary screening, could be the brand-new choice of sensing units for medical devices aimed at this kind of approach, as multiple studies have proved their usefulness for screening applications on different biological samples [1,2,3,4,5,6,7].

These works rely on previous studies on Volatile Organic Compounds (VOCs) [8,9,10,11,12,13,14,15,16,17,18,19,20], a wide field that has opened the door to research on the application of electronic noses, biosensors, and chemoresistive sensors for medical purposes [21,22,23,24,25,26,27]. The chemical markers targeted in this research have different origins, due to the various changes that tumor cell lines and masses undergo compared to their healthy counterpart:

(1) Metabolites normally emanated from cellular breath [8,9,10] and lipid membrane peroxidation VOCs (in both cell and blood tests) [11,12,13].
(2) Vascular endothelial growth factors given from neoplasm metastatization [14,15,16,17] and wastes from destroyed circulating tumor cells (blood tests only) [18,19,20].

In this study, we compared measures from human biological samples (blood specimens to be precise, with new tests following a published method to verify consistency with the existing literature, and thus using them as a benchmark to which we could correlate the new field of application) and responses to other forms of tumor and cellular metabolites, obtained from cell cultures, in order to verify further possible applications of these devices on different targets, and their reliability on homogenous immortalized and tumor cell cultures. Various different sensors were tested on a collection of blood samples, both from healthy and tumor affected individuals (colorectal and gastric cancer) ranging between 21 and 87 years of age, and on cell cultures (fibroblasts, human embryonic kidney cells (HEK-293) and Chinese hamster ovary cells (CHO)). The reason behind the diversification of the tumors tested was the will to verify a possible coherence between sensor responses and different neoplasms. Their responses were compared to recognize recurrent patterns from which the two populations could be distinguished, and to see if common trends could appear between the different applications, with both goals verified at the end of the work.

## 2. Materials and Methods

### 2.1. Blood Sampling

Blood specimens were harvested in the Hospital of Cona, Ferrara, Italy. The protocol and the informed consent form required for the research trial were presented, accepted, and retrospectively registered from the Ethical Committee of the District of Ferrara, with trial number 170, 484 (13 July 2017). Blood was collected with the standard medical sampling technique: drawing it from the arm veins of the individuals. The blood was classified between two different donor groups:

(1) Tumor affected individuals, more specifically, patients with colorectal cancer and stomach cancer.
(2) Healthy individuals (control group).

Blood specimens were poured in collection tubes (7 mL), leaving 1 cm of headspace in every collection tube. The test tubes were in a vacuum, thus there was no headspace air contaminating the samples before their opening. The saturated vapors, composing most of the VOCs measured from the devices, were in equilibrium with blood in the liquid phase. To perform a test, the blood tubes were quickly opened and the sample was rapidly poured in a single-use, sterile container and placed inside the specimen chamber. All test tubes contained an anticoagulant agent in order to prevent coagulation in the samples, called K3-EDTA (tripotassium ethylenediaminetetraacetic acid). This standard anticoagulant (2004, European Standard EN 14820; and German Standard DIN ISO 6710 [28,29]) was already added to the test tubes from the manufacturers, in order to inhibit the natural coagulation of blood samples by steadily and irreversibly chelating (binding) calcium ions, thus preventing blood from clotting. Its effects on blood samples have been widely studied for the concentrations standardly added to the test tubes, showing no substantial biological or chemical variations in the samples, nor of the volatile chemicals exhaled from them [30,31,32]. There was not any further dilution nor alteration of the collected samples.

The data set formed during this part of the work, and analyzed with the aid of the sensors array below mentioned, is composed as follows:

- Total: 15 samples collected:
  ▫ Healthy: 8
  ▫ Tumor affected: 7
  with the following anagraphical distribution.
- Females: 4
  ▫ Healthy: 1
  ▫ Tumor affected: 3
- Males: 11
  ▫ Healthy: 7
  ▫ Tumor affected: 4

between the ages of 21 and 87 years old.

### 2.2. Cell Cultures Preparation

The following immortalized cell lines were studied in this work:

(1) Fibroblasts, derived from a primary, healthy human specimen culture, playing the role of control group.
(2) HEK 293 cells, derived from human kidney fetal cells, and immortalized with an adenovirus.
(3) CHO, immortalized Chinese hamster ovary cells.

The reason behind the use of multiple cell kinds was to test if sensors could distinguish different types between them, and not just the distinction between healthy and immortalized/tumor cells.

Immortalized cells are mutant strains of the original biologically normal ones from which they are originated, and share the following properties:

(1) Loss of the former biological function previously carried in the organism that hosted them.
(2) Continuous reproduction until nutrients and free space to reproduce are no longer available (usually 2D expansion, but for some strains closer to real tumors they also expand in 3D masses), making them closely related to neoplasms in living beings, sharing the same behavior, genesis, and metabolic mutation.

To follow the cell cultures’ evolution over time, a visible ruse was taken as an advantage. In fact, how the breeding ground color changed depended on the incubation time thanks to pH indicators melted in the Dulbecco’s Modified Eagle’s Medium (DMEM, a standard fluid used as biological breeding ground) for this purpose (the longer the incubation time, the more it turns from pinkish to yellow because of the acidification of the breeding ground itself), proving the growth of the population (visible also through the optical microscope) due to the consumption of nutrients and the increasing concentration of wastes (diluted cellular metabolites).

Each test was realized with three Petri dishes, in order to increase the emanation surface, hosted in a sterile tripod structure. The samples’ preparation has been carried out as follows:

(1) Each Petri dish (diameter =3.5 cm) was filled with 1.5 mL DMEM (high glucose) without fetal bovine serum, with various antibiotics added (penicillin, streptomycin, and glutamine) to prevent contaminations from microorganisms that could harm the cells.
(2) Fetal bovine serum was added in such a quantity to reach 10% of the total volume of the finished breeding ground.

Cells were plated in the Petri dishes with the same starting concentrations (which was estimated using a gridded glass and the optical microscope): ~250,000 units. From these, the cells were left in groups of three Petri dishes each, in the incubator (kept at 5% CO_2_ as standard preventive measure to avoid contamination from bacteria, at a temperature of 37 °C) at the same time, and then tested at different set hours and days, so that the incubation time for each group was from 4 to 48 h. For all cell samples, these two parameters were critical to define both their changes and state of proliferation during the test, and thus the increase of the plated cells’ population.

### 2.3. Sensors Technology

Chemoresistive sensors are devices capable of converting chemical or physical quantities (like gas concentration or light wavelength) into an electric signal, occurring due to the reactions happening in the material due to the measured phenomenon (oxidation and reduction due to the gaseous analytes which react with the semiconductor surface), and thus changing the electrical properties (resistance/conductance) of the sensing material. The most important solids that display semiconductor properties are silicon, germanium, and compounds of gallium, followed by metal oxides (like tin oxide, titanium oxide, zinc oxide and their doped versions, used in this work) and non-metal oxide materials (like tin sulfide), widely used in electronic devices, nanostructures, integrated circuits, lasers, and so on. All the sensors used in this work are nanostructured, which means that the geometry of the grains composing them spans tens of nanometers. The reason for this choice of manufacture lays behind the fact that this property increases the sensitivity of the sensors, since when the average diameter of the grains is close to the dimension of the depletion region, nanometric phenomena occur, like unpinning of the Fermi level and flattening of the band bending, resulting in an intrinsic increase in the sensing material performances [33]. Each sensor is composed by a sensing film of semiconductor material (metal oxides or non-metal oxides), printed on a substrate crafted from sintered alumina. This substrate hosts a platinum heater connected to two of the electrodes present on one face of the unit, while on the other face interdigitated gold contacts are inserted. These gold contacts are the same on which the semiconductor paste is printed to close the circuit, acting as variable resistors to the presence of the gas analytes. The dimensions of this sensitive layer (substrate and semiconductor film) are 2 mm in length and 2 mm in width, while their thickness spans around 330 μm. The voltage drop is read by an inverting operational amplifier, integrated to dedicated electronics to which each sensor is connected by a custom 4 pins gold support. The connection between the gold electrodes on the substrate and these supports is performed by bonding gold wires. The resulting sensing units, bonded as described above, are 18 mm in height and 7 mm in diameter each. Since every sensor is prepared, pasted, and bonded with the exact same procedure, and the custom electronics printed on each dedicated PCB (Printed Circuit Board) is exactly the same for all of them, the design factor is normalized for all the sensing units, and does not affect their performances differently.

Table 1 shows the sensors (labeled with their given laboratory inventory name, which does not always strictly refer to their chemical composition) that were used for the detection of the tumor markers emanated from human blood samples, while these last mentioned were kept at room temperature:

while Table 2 shows the ones used to test the immortalized cell lines:

Each sensor was put to its best working temperature, defined by laboratory tests (carried out in the laboratory of sensors of the Department of Physics and Earth Sciences, University of Ferrara) on the tumor markers previously studied, chosen from the literature [1,2,3,6]. The variety of sensors chosen lays upon the will to verify if chemoresistive sensors can detect variations as a whole technology, or if only some particular semiconductors can be used for screening/detection purposes, and to visualize possible correlations between their response behaviors. Further details in the sensors’ functioning, the differences between the various semiconductor materials, and their characterizations can be found in literature [1,2,3,4,5,6,7,33,34,35,36,37,38,39,40,41,42,43,44,45]. The responses are standardly defined as the average value between three output voltages measured by the sensor from the same sample, as shown in the following formula [6]:

(1) $$R=\;\frac{\frac{V_{\mathrm{sensA}}}{V_{0A}}+\frac{V_{\mathrm{sensB}}}{V_{0B}}+\frac{V_{\mathrm{sensC}}}{V_{0C}}}{3}$$

having:

$V_{\mathrm{sensA},B,C}$ potential measured from the sensor at the exposure to the chemical markers of interest,

$V_{0A,B,C}$ potential measured from the sensor in a resting state, having only environmentally filtered air flowing into the sensor chambers.

The volatile compounds exhaled were carried by the flow of filtered environmental air (to avoid contaminations and moist alteration) through the sensor chambers, where they reacted with the semiconductor film and generated the potential difference measured and used to obtain our response R.

### 2.4. Sensing Device

The sensors were hosted into a patented device [national patent number: 102015000057717], SCENT B1, already presented in literature [6], during previous research on tumor markers from blood samples. The prototype consists of a hydraulic system, powered by an internal pump inflating filtered air (using carbon and anti-bacterial 0.2 μm filters on the teflon of aspiration), that leads the emanations of the specimens analyzed from the dedicated chamber where it is hosted to the gas sensors chambers, where standard SHT11 sensors are also hosted to verify the stability of temperature and humidity conditions (29 °C and 38%, respectively). Tests were carried out in a thermostatic chamber. As already proven in the previous publication, the resulting air flux is stable and laminar [6]. Once the exhalations reach the sensors chambers, they react with them, thus giving the responses that the software written in the Labview^®^ language visualize and register for the final data analysis.

## 3. Results

**Figure 1:**
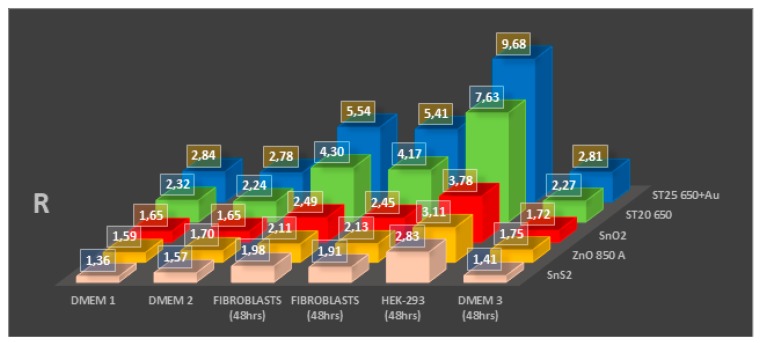
Test results from ST25 650+Au, ST20 650, SnO2, ZnO 850, and SnS2 sensors on the Dulbecco’s modified Eagle’s medium (DMEM) breeding ground, fibroblasts, and human embryo kidney cell (HEK-293) samples, incubated for the same time (48 h).

**Figure 2:**
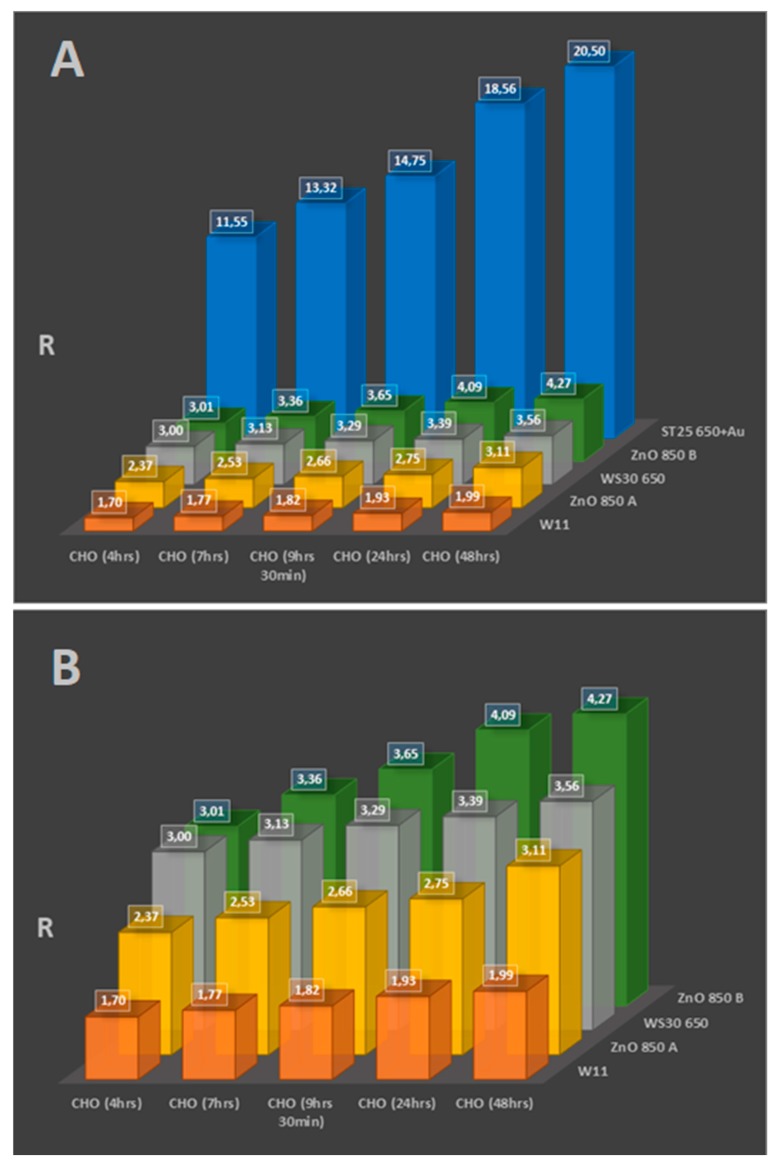
(**A**) Test results from ST25 650+Au, two ZnO 850, WS30 650, and W11 sensors on Chinese hamster ovary (CHO) cell samples incubated at different times. (**B**) A zoom on the previous tests, to better appreciate which sensor responses show trends following the different concentration in the cell cultures, due to their different incubation time.

**Figure 3:**
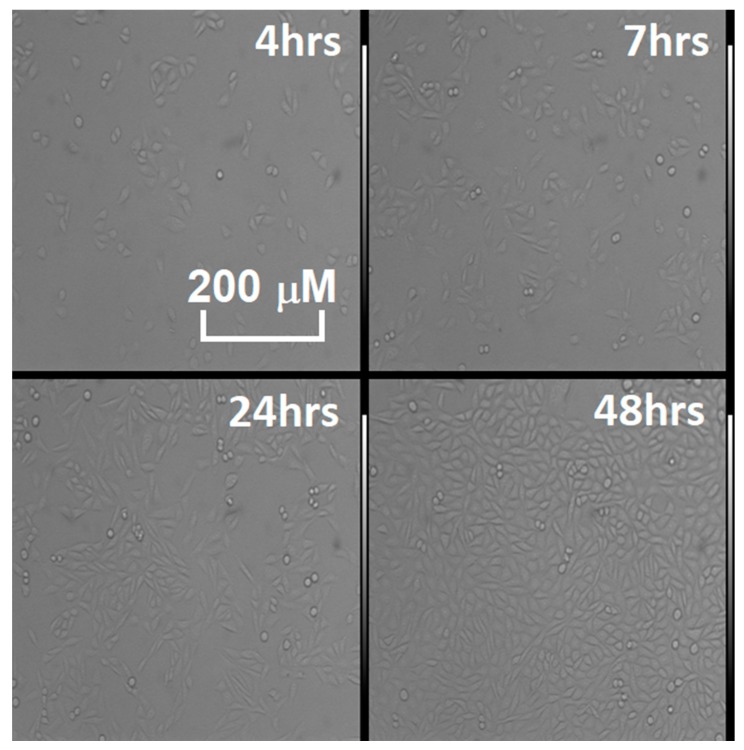
Time lapse of the proliferation in the various CHO cell cultures tested in Figure 2, observed through the optical microscope (model: TE 300, Nikon magnification 10X). The living, plated cells are the elongated semitransparent corpuscles, while the dead ones are the round shapes, floating in the breeding ground.

Figure 1 and Figure 2, Table 3 and Table 4 show the results of cell tests, complete with sensors’ response values. While most of the sensors proved to be able to distinguish between breeding grounds, healthy cells (which were not only tested multiple times, but also on multiple platings as shown in Figure 1, to ensure repeatability on the background emanations given from the DMEM), and immortalized cells, not all showed appreciable variations in their responses to discriminate between cells with different incubation times (that result in different cell population numerosities, as shown in Figure 3). Still, the results are really encouraging, with high responses from some sensors and wide differences between different cell types and incubation times.

**Figure 4:**
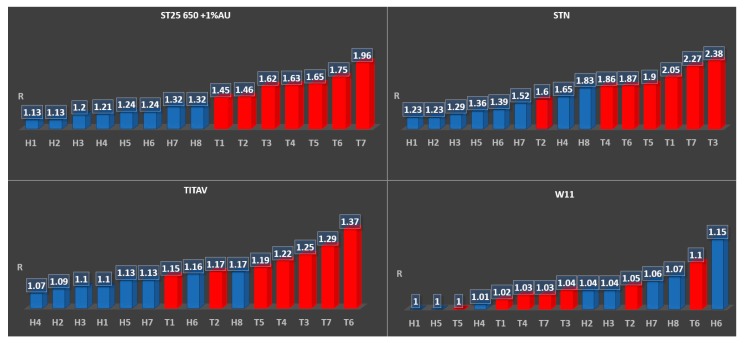
Test results from ST25 650+Au, STN, TiTaV, and W11 on multiple blood samples.

As in Figure 4, three out of four sensors showed a recognizable trend (highlighted by the labelling—H for healthy samples, T for tumor affected—and the different colors), correlating the amplitude of the response with the markers of oncological interest, even if the responses were quantitatively smaller compared to the cell tests.

## 4. Discussion

Overall, the following points have been verified during the cell tests:

(1) (Even if a cell culture biologically does not have a fast metabolism, like fibroblasts, nanostructured sensors can distinguish its activity from the hosting breeding ground.
(2) Immortalized cells have higher responses than normal cells, linked to their faster and erratic metabolism, which ends up generating greater amounts of reactive volatile metabolites, as verified multiple times in the literature [8,9,10,11,12,13,14,15,16,17,18,19,20,21,22]. This was both observed once all cell cultures were at confluence (filling the hosting Petri dish bottom completely, as shown from the responses in Figure 1 after 48 h of incubation) and during the growth of the culture populations (by comparison of the responses amplitude between Figure 1 and Figure 2, Table 3 and Table 4).
(3) The temporal evolution of metabolite production is evident, with contact inhibition (the phenomenon in which cells slow their metabolites once nutrients and space for mitosis in their surroundings starts to run out) starting at around 24 and 48 h passed without cleaning the Petri dishes by changing the breeding ground. This means that it is possible to monitor the evolution of a mass of cells (such as tumors) by studying how many metabolites they have produced over time, and monitor their vitality as well, as highlighted from results on Figure 2A,B when linked to the optical microscope pictures from Figure 3.

Concerning the blood tests, three sensors (TiTaV, STN, and ST25 650+Au) showed good capability to distinguish between healthy and tumor affected donors just from the amplitude of their responses (Figure 4, Table 5 and Table 6), even with the incapacity shown in sensors STN and TiTaV to fully distinguish patients T1 and T2 from healthy specimens. This depends on the fact that smaller and less aggressive tumors emanate smaller quantities of different metabolites and VOCs when compared to more aggressive or bigger neoplasms, as was proven in both the histological exam (which confirmed the different size and aggressiveness of the tumoral formations) and by comparison with ST25 650+Au, which instead could distinguish them as the less polluting cancers for the circulatory system by their mere observed responses. This is a huge confirmation of the work carried out on these particular sensors, since those materials were explicitly chosen from the assessments that the team could finalize from previous works [1,2,3,4,5,6,7], sealing the reliability of some semiconductors for neoplasms screening in vitro applications.

## 5. Conclusions

The study verified the correlation between tumor emanations (due to their internal changes when compared to healthy cells) and chemoresistive sensor responses; their response amplitude followed the grade of degeneration of the neoplasia, both in human blood samples and in the cell culture comparison. In particular:

(1) The ST25 650+Au, ST20 650, ZnO 850, SnO2, and SnS2 sensors showed the capability to differentiate healthy and immortalized cell lines. In particular, the ST25 650+Au and ZnO 850 sensors also proved to be able to discriminate cell culture growth in population over time just from their response amplitude.
(2) The TiTaV, STN, and ST25 650+Au sensors successfully detected the contamination of the blood stream by tumor markers, confirming their reliability after the studies already carried out in the past, showing higher responses with the worsening of the cancer degeneration.

Given these two results, we can conclude that tin and titan oxide semiconductors (and their combinations) have the properties to react successfully to be markers of oncological interest on both biological samples and direct expositions to cell lines, validating their application as fast responding materials for oncological screening purposes.

Our team is now gathering further data, from different cell types and tumor affected patients also, to increase the statistic pool and try PCA and machine learning approaches (as already carried out in previous studies [6,7]) on these new kinds of neoplasm, in order to visualize better defined patterns and program recognition algorithms from the resulting analysis. This comes with the following goals:

(1) To develop a more complete “odor fingerprint database” for tumoral cell lines, and to observe in real time the exhalations of cell metabolites. This will aim to follow cell line vitality while adding drugs for oncological treatment on their breeding ground, thus verifying their effectiveness for pharmacology studies in real time.
(2) To use these arrays as active cores for post-screening devices, aimed at giving fast responses from simple blood sampling for the follow-up of patients that need surgeries to heal from malignant neoplasms, giving physicians new reliable systems with which the existing medical protocols could be integrated.

Further tests will also be carried out on different sensing materials, testing more noble metal decorations in place of gold (like palladium or platinum), as well as different metal and non-metal oxide semiconductors, to verify if more exotic substances could distinguish tumor markers better than tin and titanium oxides.

## Figures and Tables

**Table 1:** Sensors used for the blood tests, their working temperature, a brief description of their composition, and references to other works in which they are described in depth and their characterizations are published.

Sensors for Blood Tests			
Sensor	Working Temperature (°C)	Composition	References
W11	350	Pure tungsten oxide	[34]
TiTaV	450	Titanium, tantalum and vanadium oxides	[35]
STN	450	Tin, titanium and niobium oxides	[36,37,38,39]
ST25 650+Au	450	Tin and titanium (25%) oxides, decorated with gold (1%)	[6,36,38]

**Table 2:** Sensors used for cell tests, their working temperature, a brief description of their composition, and references to other works in which they are described in depth and their characterizations are published.

Sensors for Cell Tests			
Sensor	Working Temperature (°C)	Composition	References
W11	350	Pure tungsten oxide	[34]
WS30 650	350	Tungsten and tin (30%) oxides	[2,40]
ZnO 850 A	450	Pure zinc oxide	[6,41]
ST25 650+Au	450	Tin and titanium (25%) oxides, decorated with gold (1%)	[6,36,38]
ST20 650	450	Tin and titanium (20%) oxides	[36,38]
SnO2	450	Pure tin oxide	[6,36,38]
SnS2	300	Tin sulfide	[42]
ZnO 850 B	350	Pure zinc oxide	[6,41]

**Table 3:** Data, average values, and standard deviations from cell cultures tests shown in Figure 1.

	ST25 650+Au	ST20 650	SnO2	ZnO 850 A	SnS2
DMEM1	2.82	2.32	1.66	1.59	1.33
	2.83	2.35	1.64	1.62	1.37
	2.86	2.29	1.65	1.56	1.38
AVERAGE VALUE	2.84	2.32	1.65	1.59	1.36
STANDARD DEVIATION	0.02	0.02	0.01	0.02	0.02
DMEM2	2.78	2.20	1.65	1.69	1.55
	2.74	2.23	1.63	1.70	1.56
	2.81	2.30	1.66	1.72	1.60
AVERAGE VALUE	2.78	2.24	1.65	1.70	1.57
STANDARD DEVIATION	0.03	0.04	0.01	0.01	0.02
FIBROBLASTS (48 h)	5.51	4.33	2.50	2.11	2.04
	5.56	4.29	2.51	2.09	2.00
	5.54	4.27	2.47	2.12	1.91
AVERAGE VALUE	5.54	4.30	2.49	2.11	1.98
STANDARD DEVIATION	0.02	0.02	0.02	0.01	0.05
FIBROBLASTS (48 h)	5.42	4.17	2.45	2.12	1.97
	5.41	4.14	2.42	2.15	1.89
	5.39	4.20	2.48	2.13	1.87
AVERAGE VALUE	5.41	4.17	2.45	2.13	1.91
STANDARD DEVIATION	0.01	0.02	0.02	0.01	0.04
HEK-293 (48 h)	9.67	7.67	3.78	3.13	2.75
	9.70	7.60	3.75	3.11	2.89
	9.68	7.63	3.82	3.09	2.86
AVERAGE VALUE	9.68	7.63	3.78	3.11	2.83
STANDARD DEVIATION	0.01	0.03	0.03	0.02	0.06
DMEM3 (48 h)	2.81	2.24	1.73	1.77	1.41
	2.79	2.30	1.70	1.75	1.43
	2.82	2.26	1.74	1.73	1.38
AVERAGE VALUE	2.81	2.27	1.72	1.75	1.41
STANDARD DEVIATION	0.01	0.02	0.02	0.02	0.02

**Table 4:** Data, average values, and standard deviations from cell cultures tests shown in Figure 2A,B.

	ST25 650+Au	ZnO 850 A	WS30 650	ZnO 850 A	W11
CHO (4 h)	11.52	3.03	3.00	2.35	1.70
	11.55	2.98	3.02	2.41	1.88
	11.58	3.02	2.98	2.34	1.53
AVERAGE VALUE	11.55	3.01	3.00	2.37	1.70
STANDARD DEVIATION	0.02	0.02	0.02	0.03	0.14
CHO (7 h)	13.30	3.36	3.15	2.55	1.59
	13.34	3.35	3.11	2.52	1.77
	13.32	3.37	3.13	2.53	1.94
AVERAGE VALUE	13.32	3.36	3.13	2.53	1.77
STANDARD DEVIATION	0.02	0.01	0.02	0.01	0.14
CHO (9 h 30 min)	14.73	3.62	3.28	2.66	1.80
	14.76	3.65	3.33	2.64	1.88
	14.75	3.67	3.25	2.67	1.79
AVERAGE VALUE	14.75	3.65	3.29	2.66	1.82
STANDARD DEVIATION	0.01	0.02	0.03	0.01	0.04
CHO (24 h)	18.56	4.12	3.35	2.73	1.93
	18.58	4.05	3.36	2.71	1.87
	18.53	4.09	3.45	2.80	2.00
AVERAGE VALUE	18.56	4.09	3.39	2.75	1.93
STANDARD DEVIATION	0.02	0.03	0.04	0.04	0.05
CHO (48 h)	20.49	4.27	3.51	3.09	1.95
	20.50	4.24	3.60	3.08	2.04
	20.51	4.30	3.56	3.16	1.97
AVERAGE VALUE	20.50	4.27	3.56	3.11	1.99
STANDARD DEVIATION	0.01	0.02	0.04	0.04	0.04

**Table 5:** Data, average values, and standard deviations from blood tests on healthy samples shown in Figure 4.

	ST25 650+Au	STN	TiTaV	W11
H1	1.12	1.25	1.10	1.00
	1.15	1.20	1.10	0.99
	1.12	1.24	1.09	1.00
AVERAGE VALUE	1.13	1.23	1.10	1.00
STANDARD DEVIATION	0.01	0.02	0.00	0.00
H2	1.14	1.23	1.11	0.99
	1.15	1.22	1.09	1.05
	1.11	1.24	1.08	1.07
AVERAGE VALUE	1.13	1.23	1.09	1.04
STANDARD DEVIATION	0.02	0.01	0.01	0.03
H3	1.22	1.30	1.12	1.02
	1.19	1.31	1.11	1.02
	1.19	1.27	1.08	1.08
AVERAGE VALUE	1.20	1.29	1.10	1.04
STANDARD DEVIATION	0.01	0.02	0.02	0.03
H4	1.21	1.64	1.09	1.03
	1.23	1.67	1.04	1.01
	1.18	1.65	1.07	1.00
AVERAGE VALUE	1.21	1.65	1.07	1.01
STANDARD DEVIATION	0.02	0.01	0.02	0.01
H5	1.26	1.38	1.14	0.99
	1.23	1.34	1.13	1.01
	1.22	1.36	1.12	1.01
AVERAGE VALUE	1.24	1.36	1.13	1.00
STANDARD DEVIATION	0.02	0.02	0.01	0.01
H6	1.25	1.40	1.19	1.15
	1.23	1.37	1.14	1.11
	1.23	1.41	1.15	1.20
AVERAGE VALUE	1.24	1.39	1.16	1.15
STANDARD DEVIATION	0.01	0.02	0.02	0.04
H7	1.33	1.52	1.12	1.04
	1.32	1.51	1.15	1.06
	1.30	1.52	1.12	1.07
AVERAGE VALUE	1.32	1.52	1.13	1.06
STANDARD DEVIATION	0.01	0.00	0.01	0.01
H8	1.32	1.85	1.19	1.11
	1.30	1.82	1.15	1.05
	1.34	1.81	1.17	1.05
AVERAGE VALUE	1.32	1.83	1.17	1.07
STANDARD DEVIATION	0.02	0.02	0.02	0.03

**Table 6:** Data, average values and, standard deviations from blood tests on tumor affected samples shown in Figure 4.

	ST25 650+Au	STN	TiTaV	W11
T1	1.47	2.03	1.13	1.03
	1.43	2.04	1.18	1.04
	1.44	2.07	1.15	0.98
AVERAGE VALUE	1.45	2.05	1.15	1.02
STANDARD DEVIATION	0.02	0.02	0.02	0.03
T2	1.46	1.62	1.18	1.08
	1.46	1.61	1.13	1.02
	1.47	1.58	1.19	1.04
AVERAGE VALUE	1.46	1.60	1.17	1.05
STANDARD DEVIATION	0.00	0.02	0.03	0.02
T3	1.64	2.35	1.26	1.04
	1.62	2.41	1.22	1.06
	1.59	2.38	1.27	1.03
AVERAGE VALUE	1.62	2.38	1.25	1.04
STANDARD DEVIATION	0.02	0.02	0.02	0.01
T4	1.66	1.89	1.20	1.05
	1.60	1.85	1.23	1.03
	1.62	1.84	1.22	1.01
AVERAGE VALUE	1.63	1.86	1.22	1.03
STANDARD DEVIATION	0.02	0.02	0.01	0.02
T5	1.65	1.92	1.20	1.01
	1.66	1.89	1.19	1.03
	1.64	1.90	1.18	0.97
AVERAGE VALUE	1.65	1.90	1.19	1.00
STANDARD DEVIATION	0.01	0.01	0.01	0.02
T6	1.76	1.88	1.37	1.09
	1.75	1.88	1.38	1.08
	1.73	1.85	1.37	1.13
AVERAGE VALUE	1.75	1.87	1.37	1.10
STANDARD DEVIATION	0.01	0.01	0.00	0.02
T7	1.96	2.27	1.30	1.02
	1.99	2.28	1.27	1.04
	1.94	2.27	1.31	1.02
AVERAGE VALUE	1.96	2.27	1.29	1.03
STANDARD DEVIATION	0.02	0.00	0.02	0.01
